# Computational Design, Synthesis, and Pharmacological Evaluation of Naproxen-Guaiacol Chimera for Gastro-Sparing Anti-Inflammatory Response by Selective COX2 Inhibition

**DOI:** 10.3390/molecules27206905

**Published:** 2022-10-14

**Authors:** Pottathil Shinu, Manu Sharma, Girdhari Lal Gupta, Somdutt Mujwar, Mahmoud Kandeel, Manish Kumar, Anroop B. Nair, Manoj Goyal, Purna Singh, Mahesh Attimarad, Katharigatta N. Venugopala, Sreeharsha Nagaraja, Mallikarjun Telsang, Bandar E. Aldhubiab, Mohamed A. Morsy

**Affiliations:** 1 Department of Biomedical Sciences, College of Clinical Pharmacy, King Faisal University, Al-Ahsa 31982, Saudi Arabia; 2 Department of Chemistry, National Forensic Sciences University Delhi Campus, New Delhi 110085, India; 3 Department of Pharmacology, School of Pharmacy and Technology Management, SVKM’s NMIMS University, Shirpur 425405, India; 4 Chitkara College of Pharmacy, Chitkara University, Rajpura 140401, India; 5 Department of Biomedical Sciences, College of Veterinary Medicine, King Faisal University, Al-Ahsa 31982, Saudi Arabia; 6 M.M College of Pharmacy, Maharishi Markandeshwar (Deemed to be University), Ambala 133201, India; 7 Department of Pharmaceutical Sciences, College of Clinical Pharmacy, King Faisal University, Al-Ahsa 31982, Saudi Arabia; 8 Department of Anesthesia Technology, College of Applied Medical Sciences in Jubail, Imam Abdul Rahman Bin Faisal University, Jubail 35816, Saudi Arabia; 9 Department of Physiology, College of Medicine, Saint James School of Medicine, The Valley 3872, Anguilla; 10 Department of Biotechnology and Food Science, Faculty of Applied Sciences, Durban University of Technology, Durban 4000, South Africa; 11 Department of Pharmaceutical Chemistry, Vidya Siri College of Pharmacy, Off Sarjapura Road, Bangalore 560035, India; 12 Department of Surgery, College of Medicine, King Faisal University, Al-Ahsa 31982, Saudi Arabia; 13 Department of Pharmacology, Faculty of Medicine, Minia University, El-Minia 61511, Egypt

**Keywords:** naproxen, guaiacol, chimera, S-naproxen-4-allylguaiacol, anti-inflammatory, biomedical, treatment, gastro-sparing

## Abstract

The 4-allyl guaiacol is a natural phenolic molecule that has been widely studied for its antioxidant capacity against reactive-oxygen-species-mediated cellular damage. Therefore, we hypothesized that concomitant use of an antioxidant and NSAID may decrease the risk of gastrointestinal toxicity and make the therapy safer. To address the gastrointestinal toxicity of conventional NSAIDs, a new S-naproxen-4-allyl guaiacol chimera (MAS-1696) was computationally developed, chemically synthesized, and tested for anti-inflammatory effectiveness and gastrointestinal safety. The inhibitory potency of MAS-1696 tested against cyclooxygenase-2 (COX2), 15-lipoxygenase-2 (15-LOX2), and lipoxygenase-5 (5-LOX) in vitro revealed a stronger inhibition of COX2. Furthermore, the MAS-1696 chimera increased the COX selectivity index by 23% as compared to the parent compound naproxen, implying higher efficacy and gastric safety. In vivo data showed that MAS-1696 was less likely to cause gastrointestinal harm than naproxen while also exerting anti-inflammatory and analgesic effects equivalent to or superior to naproxen. In conclusion, MAS-1696 is orally active, bio-labile, and crystalline, making it a medication that may be administered orally.

## 1. Introduction

Non-steroidal anti-inflammatory drugs (NSAIDs) such as S-naproxen are one of the most extensively prescribed medicines for the treatment and management of inflammatory conditions. With the rise in inflammatory disorders such as arthritis, chronic pain, and muscular and sports injuries, NSAID prescriptions and consumption are gradually increasing. Long-term usage of NSAIDs has been linked to serious adverse effects such as erosions, strictures, ulcers, bleeding, and perforation within the gastrointestinal (GI) tract [1]. To address these concerns, a series of selective COX2 inhibitors such as Rofecoxib and Celecoxib was clinically approved in the early 1990s, with remarkable anti-inflammatory potencies and low gastric toxicity. However, the early enthusiasm for selective COX2 inhibitors has waned as a result of the identification of significant cardiovascular adverse effects from their prolonged use [2]. Although the etiology, as well as the pathogenesis, of NSAID-induced gastric toxicity is not fully understood [3,4,5,6,7], various studies have shown that gastric injury depends on several factors such as the pKa and lipid solubility (log P) of NSAIDs. The majority of NSAIDs are weak acids because of a free acidic functional group in their structures. The acidic property and lipophilic characteristic of heterocyclic carboxylic acids are co-dependent and the free carboxylic group in NSAIDs influences the water solubility and imparts detergent-like properties. These surfactants-like characteristics of NSAIDs are responsible for contact with the membrane phospholipids and determine their degree of internalization within gastric cells and the extent of intracellular ion trapping [1,8]. NSAIDs are thought to accumulate in stomach epithelial cells through a process known as ‘ion trapping’, followed by the production of reactive oxygen species (ROS) such as superoxides and hydroxyl radicals [1].

S-naproxen is a nonselective COX inhibitor having anti-inflammatory potency. The anti-inflammatory effect of S-naproxen is associated with the inhibition of both COX1 and COX2 enzymes. However, owing to poor selectivity, naproxen is associated with serious gastrointestinal toxicity. COX1 inhibition results in gastrointestinal mucous depletion, which has been linked to gastrointestinal toxicity. The mucous layer is a protective coating found in stomach tissues that prevent the epithelial gastric tissues from being auto-digested by hydrochloric acid. Depletion of this protective mucous layer in the stomach may result in tissue damage from gastric hydrochloric acid, which can lead to a variety of issues such as a gastric ulcer. Furthermore, S-naproxen contains a free carboxyl group that, when ionized, contributes to acidity, lowering the gastric pH and complicating the issue [9,10].

**Figure 1:**
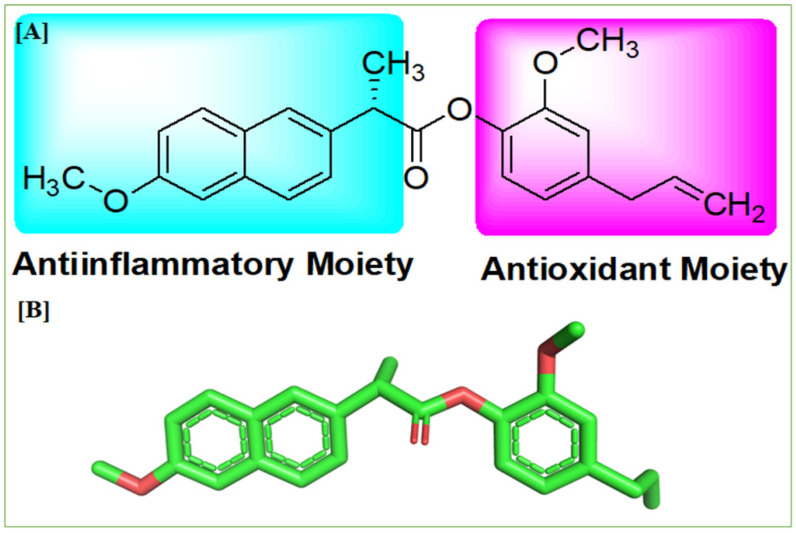
(**A**) Chemical structure of MAS-1696 (**2**). Anti-inflammatory and antioxidant scaffolds of MAS-1696 are highlighted. (**B**) Energy-minimized 3-dimensional structure of MAS-1996 (**2**).

Several studies have shown the hyperproduction of ROS in the intestines of patients receiving recurrent doses of NSAIDs, and a disproportion of essential antioxidants, resulting in oxidative damage [11,12]. The 4-allyl guaiacol is a natural phenolic molecule and has been widely studied for its antioxidant capacity against ROS-mediated cellular damage [13,14,15,16,17,18]. Therefore, we hypothesized that conjugation of 4-allyl guaiacol with naproxen may reduce the chances of gastric toxicity and result in safer therapeutics. Unfortunately, orally administered antioxidants are not sufficiently effective, due to high hydrophilicity and poor bioavailability [19,20,21,22,23,24,25,26,27,28,29,30]. To address these issues, we created a chimera composed of two distinct therapeutic compounds with complimentary pharmacological actions and better physicochemical qualities. This chimera not only masks the free carboxylic group of NSAIDs but also acts as an antioxidant to quench free radicals following hydrolysis. This chimera offers an array of new improved anti-inflammatory molecules with exceptional gastro-sparing properties, and, at the same time, it improves the physiochemical properties of the antioxidant moiety [17,31,32,33,34,35,36,37]. In the present research, we report the computational design, synthesis, and assessment of a new S-naproxen-4-allyl guaiacol chimera (MAS-1696) for its anti-inflammatory and gastro-sparing properties (Figure 1). MAS-1696′s physicochemical properties and its likely binding mode with COX1, COX2, and 5-LOX enzymes have also been investigated.

## 2. Results

### 2.1. Molecular Docking Studies

#### 2.1.1. Ligand Design

With the intent to mask the free carboxyl group as well as to increase the selectivity of the COX2 enzyme, a conjugate compound is synthesized by the fusion of S-naproxen with the antioxidant compound guaiacol by using its carboxyl moiety (Figure 1). Being a strong antioxidant, it should be able to oxidize the free radicals responsible for gastrotoxicity and conceal the free carboxyl group, which will reduce the acidic effect.

The conjugation product of S-naproxen and guaiacol is also supposed to increase the selectivity toward the COX2 enzyme required for avoiding the undesirable mucosal depletion effect caused by the inhibition of the COX1 enzyme. The active binding site of COX2 was found to be larger when compared to that of COX1. Thus, the selectivity of the conjugated product is supposed to be much higher toward the COX2 enzyme because of its bulkiness, which may cause difficulty in its adjustment within the small binding cavity of the COX1 enzyme. Due to the aforementioned factors, a conjugation molecule was created by fusing S-naproxen and guaiacol at the carboxyl site of the former.

#### 2.1.2. Protein Targets

The conjugation product has been further evaluated by molecular docking as well as a dynamic simulation for their binding with various anti-inflammatory targets COX2, 15-LOX2, 5-LOX, and also including COX1 for predicting their selectivity based upon binding affinity and stability concerning time. The structure of COX1 consists of two chains having 553 amino acids and complexed ligand celecoxib. The structure of the COX2 enzyme consists of a dimeric structure and each chain has 551 amino acids complexed with mefenamic acid. The three-dimensional structure of 5-LOX is a dimeric structure having 691 amino acids in each chain complexed with the endogenous ligand arachidonic acid. The structure of the 15-LOX2 enzyme has two chains, each of 689 amino acids complexed with an imidazole-based inhibitor. The structure of RORγ has a single chain of 238 amino acids complexed with an imidazole-based potent inhibitor.

#### 2.1.3. Docking Outcomes

The docking methodology for each of the macromolecular targets was validated effectively as the docked conformations of all the reference ligands were flawlessly overlaid over their bioactive conformations with similar types of binding interactions. Following validation, the produced conjugated ligand and the standard drug molecule were docked using the same docking parameters. The docking results are tabulated in Table 1.

**Figure 2:**
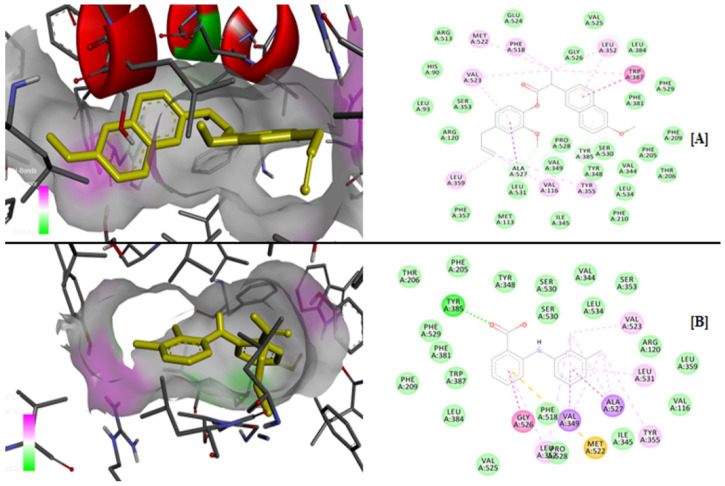
Two- and three-dimensional binding conformation of (**A**) designed chimera MAS-1696 and (**B**) standard drug S-naproxen against human COX2 enzyme. The interactions shown in pink color refer to pi interactions; the green color refers to van der Waals interactions, and light green refers to hydrogen bonding.

Analysis of docking results indicates that the designed conjugate ligand has a strong affinity for COX2 when compared to the standard drug *S*-naproxen and the reference ligand mefenamic acid present in the crystallized complex. In addition, the designed ligand has the least affinity for the COX1 enzyme when compared to the standard drug *S*-naproxen and reference co-crystallized ligand celecoxib. The binding conformation of the designed chimera and the standard drug S-naproxen is shown in Figure 2. The details of interacting residues for the designed chimera MAS-1696 against other anti-inflammatory drug targets such as 15-LOX2, 5-LOX, RORγ, and COX1 are tabulated in Table S3.

### 2.2. Molecular Dynamics Simulation

The macromolecular complex of the human COX2 receptor with the designed conjugate inhibitor was re-validated by performing MD simulation for 100 ns using Schrodinger’s Desmond tool. The macromolecular receptor has 551 residues, whereas the ligand has eight rotatable bonds and 28 heavy atoms out of a total of 52 atoms. The RMSD analysis facilitates the effective implementation of the equilibrated simulation process based on structural validation. During the simulation process, the ligand’s RMSD shows its stability concerning the macromolecular binding residues by alignment with heavy metals.

**Figure 3:**
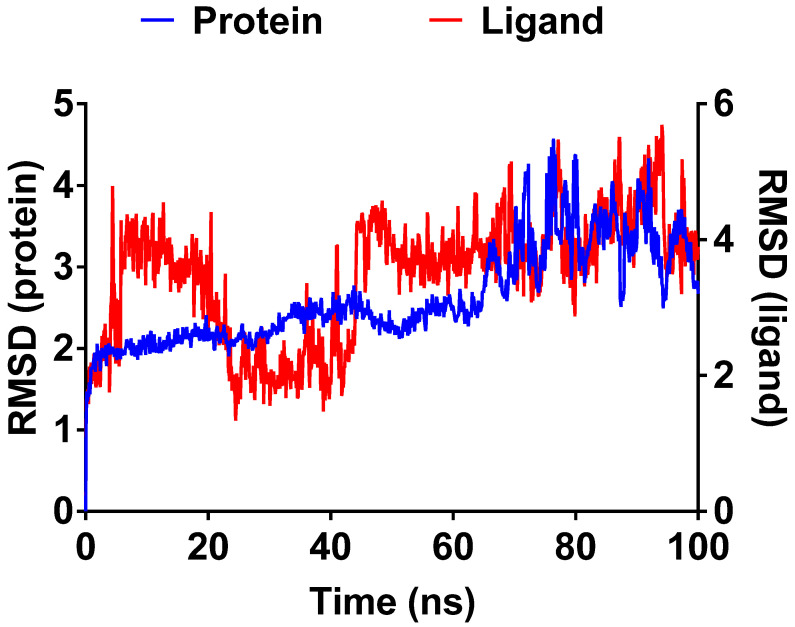
RMSD of the Cα backbone of human COX2 receptor and MAS-1696 ligand observed during the 100 ns MD simulation.

RMSD for the macromolecular residues were discovered to be within the range of 2–4.5 Å, describing that most of the residues do not move from their initial location during ligand molecule complexation. The macromolecular backbone remains stable throughout the simulation process and some of the residues fluctuate to some extent to achieve the most stabilized conformation leading to the attainment of the overall stability of the macromolecular backbone within the complex. The complex chimera-based ligand takes a couple of moves within the macromolecular cavity to adjust itself, leading to the attainment of the stabilized conformation within the macromolecular cavity. In the initial phase of the simulation, the ligand fluctuates within the range of 2–5 Å while taking certain moves to achieve the stabilized conformation followed by a little fluctuation within the range of 4–5 Å throughout the later phase of the simulation. Therefore, these ongoing vibrations while executing specific maneuvers attain the firmest confirmation within the binding cavity of the human COX2 receptor, which causes the early oscillations in the ligand’s RMSD value during 0–5 ns. The RMSD of the macromolecular complex during the 100 ns of the MD simulation is displayed in Figure 3.

**Figure 4:**
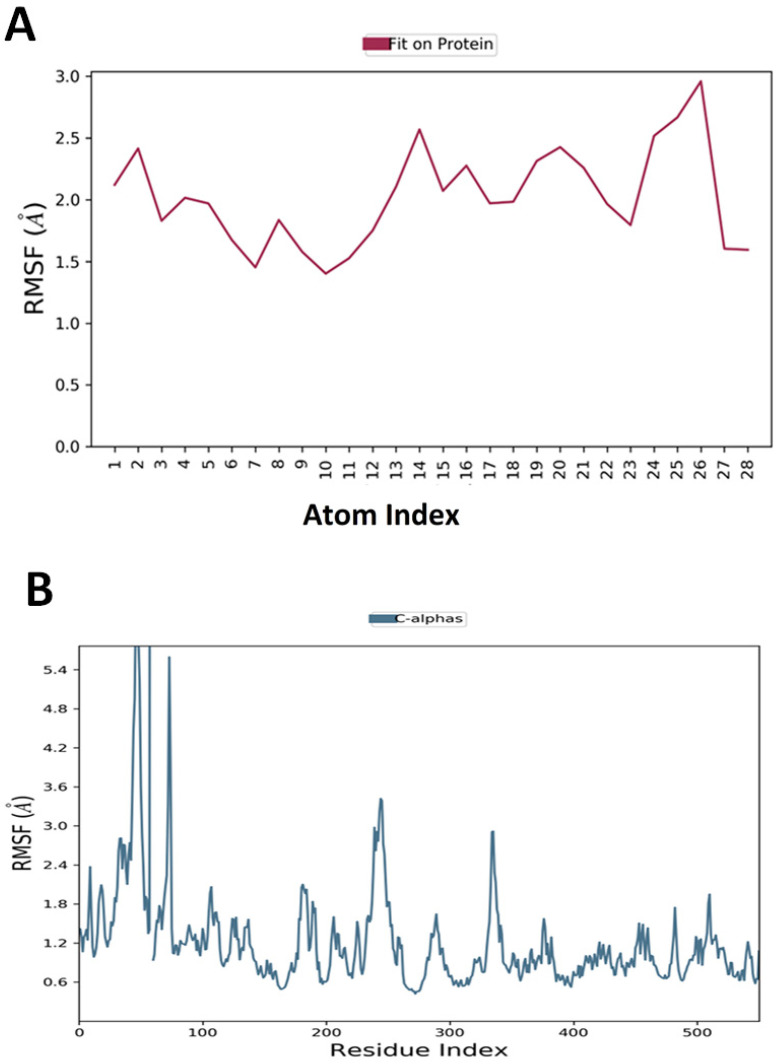
The root-mean-square fluctuation during the 100 ns timeframe of the MD simulation, the RMSF of the human COX2 receptor, and the complexed MAS-1696 ligand were measured. (**A**) The ligand RMSF. (**B**) The protein RMSF.

Except for a few terminal residues that fluctuated a bit with an RMSF value of 5.4 Å, the majority of the protein residues’ RMSF values were observed to be well within the permitted range of 1~2 Å by fluctuating within 0–6~1.8 Å. The RMSF of the human COX2 receptor and the complex conjugate ligand during the first 100 ns of the MD simulation is depicted in Figure 4. The RMSF measured for the conjugate ligand complex within the human COX2 receptor’s active binding region was determined to be within 2–3 Å throughout a 100 ns period. This demonstrates that the ligand was stabilized enough within the target macromolecule’s active site, with only minor fluctuations required to interact with the protein backbone.

**Figure 5:**
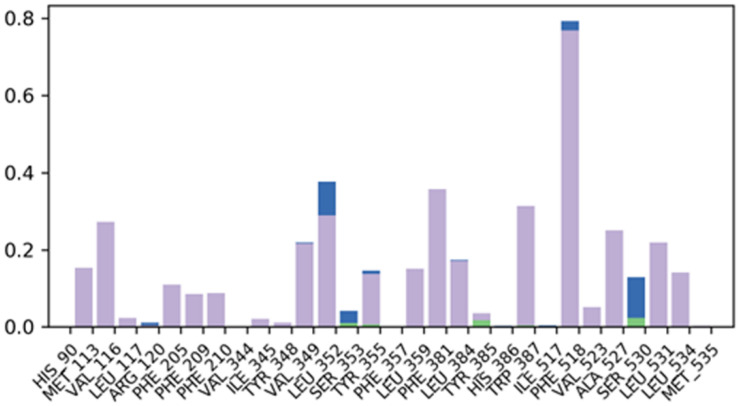
Protein–ligand contacts and detailed protein–ligand interactions were found during the 100 ns MD simulation timescale. H-bonds in green, hydrophobic interactions in slate, and water bridges in blue.

Secondary structural elements (SSE) examination throughout the simulation process indicated that it has 31.90% α-helices and 3.91% β-strands, totaling 35.81% SSE, which may be conserved for the bulk of the simulation duration. Figure 5 shows the specific protein–ligand linkages that were seen throughout the course of the whole 100 ns MD simulation. The complex ligand was shown to bind with more than eight residues consistently throughout the simulation. The protein–ligand interaction study revealed that Met113, Val116, Val349, Leu352, Phe381, Trp387, Phe518, and Ala527 were interacting with the ligand molecule throughout the 100 ns simulation.

**Figure 6:**
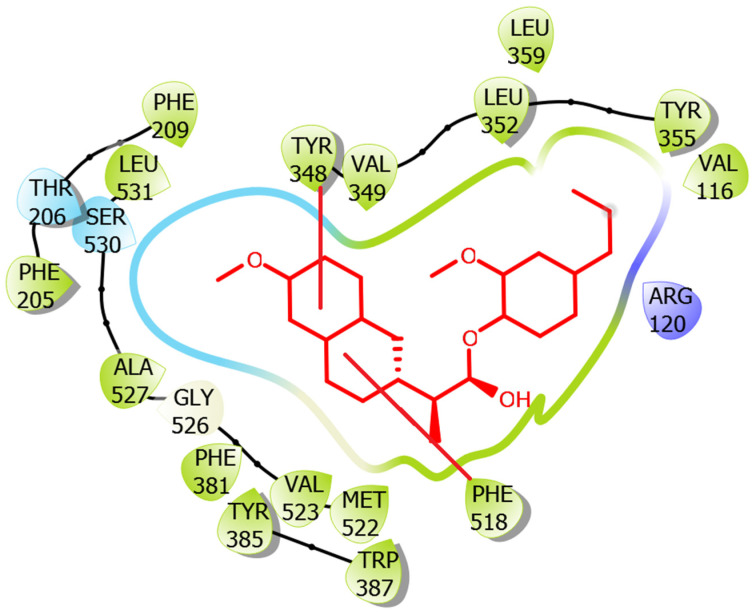
Two-dimensional ligand interactions of MAS1696 with human COX2 enzyme.

The two-dimensional interaction of the human COX2 with the designed conjugate inhibitor is shown in Figure 6. The ligand interactions showed the stacking interactions of MAS1696 with COX2 residues Tyr348 and Phe518. These interactions stabilized the binding of MAS1696 with the active site. None of intramolecular hydrogen bonding was observed during the simulation.

### 2.3. Pharmacokinetic and Toxicological Profiling

Pharmacokinetic and physicochemical features control the kinetics of the drug through the human body. These regulatory factors are essential for a drug molecule’s pharmacological expression through pharmacodynamics, as well as its toxicological characteristics. The lead compounds’ physicochemical, pharmacokinetic, and toxicological characteristics were predicted using the pkCSM webserver. Table S4 lists the physicochemical, pharmacokinetic, and pharmacodynamic parameters of the selected lead compounds.

The *S*-naproxen-based chimera ligand has most of the parameters well within the predefined range according to Lipinski’s rule of five, i.e., MW < 500, <10 HBA, <5 HBD, and LogP value. The shown physicochemical characteristics imply that the conjugated ligand has ideal pharmacokinetics for drug-like candidates. The biological barrier for xenobiotics and exogenous toxins is P-glycoprotein, whose conjugated ligand has not been shown to express in a manner similar to that of a substrate.

Except for the metabolic CYP3A4, the conjugated ligand has not demonstrated substrate-like expression for the majority of cytochrome P450 isoenzymes. The ligand repeatedly demonstrates a high inclination for excretion from biological systems and low expression across a number of toxicity pathways, including AMES, hERG I inhibition, skin sensitization, Tetrahymena Pyriformis toxicity, and minnow toxicity. Overall, the conjugated compound’s expected physicochemical, pharmacokinetics, pharmacodynamics, and toxicological characteristics all fit well within the prescribed range.

### 2.4. Chemical Synthesis

*S*-naproxen was reacted with acetyl chloride with pyridine and dichloromethane to afford a crude anhydride product, which was used in the next step without further purification. The anhydride derivative of *S*-naproxen and 4-allylguaiacolin equimolar amounts were refluxed in pyridine and, after completion of the reaction, it was processed. The solvent was distilled off and the residue was chromatographed over silica gel to give a purified final product. The structures of intermediates and the final product were elucidated by using various spectroscopic techniques (Figures S1–S4 and Table S1).

### 2.5. Pharmacological Evaluation

The in vitro inhibition potency of MAS-1696 was evaluated for COX1, COX2, and 5-LOX. The results indicated that MAS-1696 inhibits COX2 with IC_50_ of 2.92 µg/mL, whereas the potency of MAS-1696 against COX1 and 5-LOX was lower (Table 2). When compared to the parent molecule naproxen (0.85), MAS-1696 produced a greater selectivity index (1.1). This implies a 23% rise in selectivity for COX2 over COX1 by MAS-1696.

**Figure 7:**
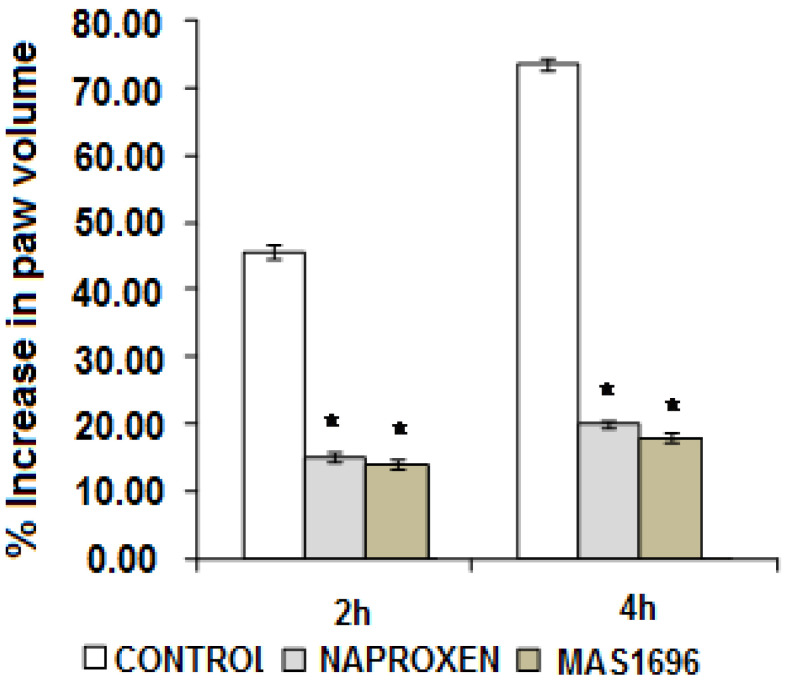
Anti-inflammatory activity of *S*-naproxen and MAS1696. * *p* < 0.05 as compared to control and naproxen. Results presented are mean ± S.E.M (*n* = 6).

**Figure 8:**
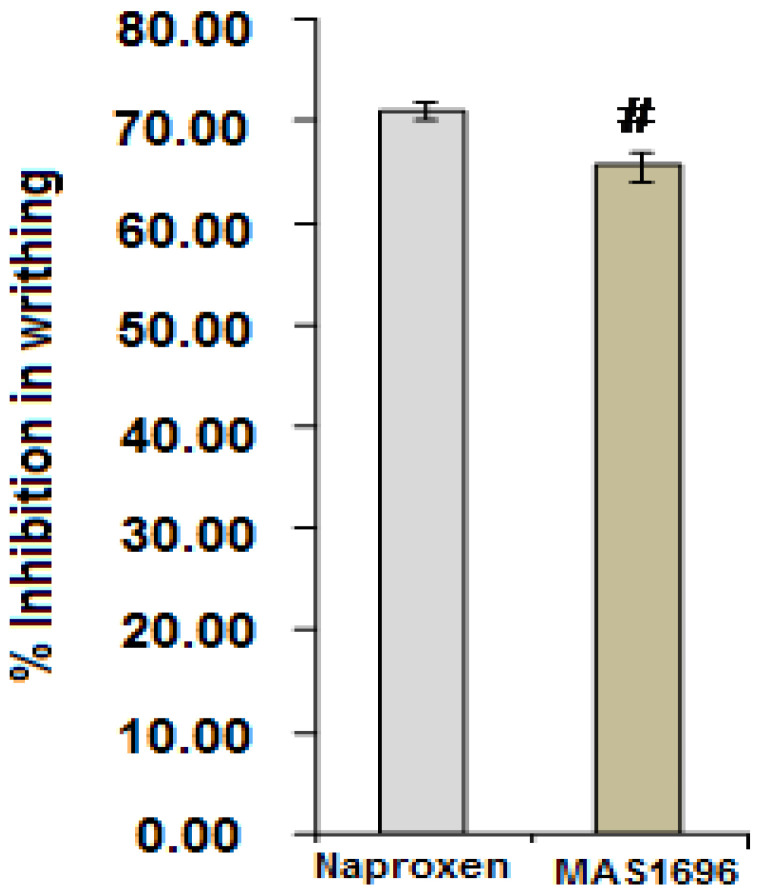
Analgesic activity of *S*-naproxen and MAS1696. # *p* < 0.05 as compared to S-naproxen. Results presented are mean ± S.E.M (*n* = 6).

In the anti-inflammatory evaluation of MAS1696, carrageenan (1% *w*/*v*) induced paw edema in the animals. Edema was inferred as the rise (%) in the volume of the right hind paw when compared to the un-injected left hind paw. There was a significant decrease in the edema and paw volume in the *S*-naproxen (15 mg/kg, p.o)-treated group when compared with the control. The results showed that MAS1696 had a better anti-inflammatory effect when compared with *S*-naproxen (*p* < 0.05) at 2 h and 4 h (Figure 7). The per se effect of promoiety 4-allyl guaiacol (15.46 mg/kg) did not demonstrate an anti-inflammatory effect. The analgesic activity of MAS1696 was evaluated against acetic-acid-induced writhing in mice, and *S*-naproxen illustrated considerable inhibition at 15 mg/kg. The equimolar dose of MAS-1696 (21.1 mg/kg) exhibited a similar analgesic effect as compared to *S*-naproxen (Figure 8). The per se effect of the 4-allyl guaiacol (15.46 mg/kg) did not demonstrate an analgesic effect.

### 2.6. Ulcer Index Examination

**Figure 9:**
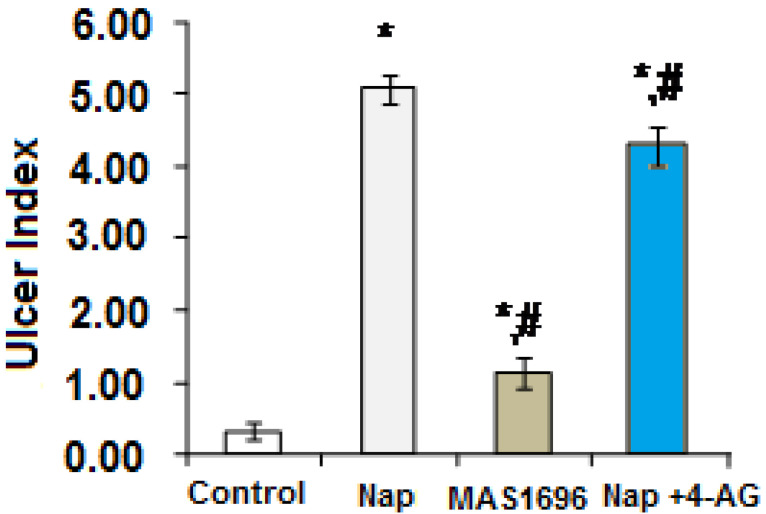
Comparative effect of *S*-naproxen (120 mg/kg), the molar equivalent of MAS-1696, and *S*-naproxen and 4-allyl guaiacol physical mixture (Nap + 4-AG) on gastric damage in the stomach. * p < 0.05 vs. vehicle-treated group; *# p* < 0.05 vs. naproxen-treat group. Results presented are mean ± S.E.M. of six animals in each group.

**Figure 10:**
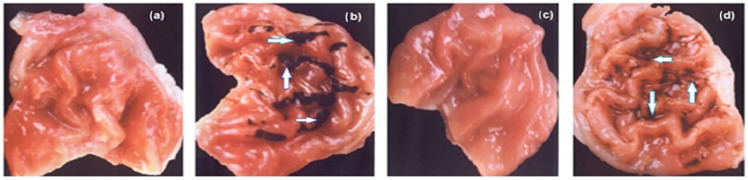
Gastrotoxic effects on the administration of (**a**) 0.5% CMC (control); (**b**) *S*-naproxen-treated group; (**c**) equimolar MAS-1696-treated group; (**d**) molar equivalent of *S*-naproxen + 4-allyl guaiacol physical mixture. Arrows indicate ulceration and lesions.

*S*-naproxen (120 mg/kg, p.o.) produced a substantial rise in ulcer score compared with the control (Figure 9). On the contrary, considerably less damage was noticed in the animals treated with the equimolar dose of MAS-1696 vs. naproxen (*p* < 0.05) and vs. Nap + 4-AG (*p* < 0.5) (Figure 9). The morphological changes in the gastric tissue before and after the administration of *S*-naproxen, MAS-1696, and the physical mixture of naproxen and 4-allyl guaiacol are depicted in Figure 10. The figure describes the significant improvement in gastric lesions under the effect of MAS-1696.

### 2.7. Determination of Distribution Coefficient (Log P)

The partition/distribution coefficient is a measure of the bioavailability of drugs and indicates the ability of the drug to cross the biological membrane. A low log *p* value may hamper the chances of the molecule being developed as a drug, as it will yield poor bioavailability. The log *p* value of MAS-1696 was determined by the shake flask method with the help of HPLC. The MAS-1696 exhibited log *p* values of 4.70 (Table 3). According to Lipinski’s rule of five, the octanol-water partition/distribution coefficient or log *p* should not be greater than 5 and the MAS-1696 obeyed Lipinski’s rule of five.

### 2.8. Chemical and Enzymatic Hydrolysis Studies

The gastrointestinal (GI) tract should not be exposed to the free acidic group of NSAIDs, as it may cause gastric ulcers and perforation of the GI wall. Therefore, MAS-1696 should be sufficiently stable to pass through the stomach conditions in the unhydrolyzed form. The MAS-1696 was treated with simulated gastric fluid for 0, 2, 5, 8, and 12 h at a pH of 2. HPLC analysis of MAS-1696 samples withdrawn after 0, 2, 5, 8, and 12 h indicated that only 0, 3.84, 9.28, 16.7, and 22.97% of MAS-1696 was hydrolyzed in a simulated gastric fluid. In other words, 100, 96.16, 90.72, 83.30, and 77.03% of MAS-1696 passed through simulated stomach conditions in the unhydrolyzed form after 0, 2, 5, 8, and 12 h, respectively (Table 3), and was chemically stable enough to be developed as a prodrug. One of the other essential characteristics of the prodrug is that it should be quickly hydrolyzed in the human plasma to disseminate the prodrug molecules. In order to study the response of MAS-1696 in human plasma, it was treated with 80% human plasma for 0, 30, and 60 min. HPLC analysis of the exposed samples indicated that MAS-1696 was completely hydrolyzed within half an hour (Table 4). Rapid hydrolysis of MAS-1696 in human plasma indicated that this compound can be successfully developed as a prodrug molecule.

### 2.9. Powder X-ray Diffraction Studies

The morphological characteristics of active pharmaceutical ingredients are important parameters for its development as an oral formulation. A solid-state morphology is specifically essential for API, as their morphological structure can have crucial effects on their blood plasma level and stability. Therefore, it is required to identify the solid-state morphology of MAS-1696. The diffraction spectrum of MAS-1696 showed that the prodrug was crystalline powder when screened in an X’Pert PRO diffractometer. The MAS-1696 showed sharp peaks at 2θ equal to 23.29°, 17.23°, 10.12°, and 9.00°. The X-ray diffraction spectrum of MAS-1696 is shown in Figure S5 and Table S2.

## 3. Discussion

Apart from the reduction of endogenous prostaglandins through suppression of the cyclooxygenase, there are a number of other causes for the gastrotoxicity of NSAIDs. The most likely mode of NSAID-induced gastropathy includes an increase in the mucosal production of pro-inflammatory cytokines, enhanced lipid peroxidation [10], and the adherence of neutrophils to the microvascular endothelium, which results in the generation of free radicals [29]. Some reports show that the free acidic function of NSAIDs enhances its aqueous solubility and also imparts surfactant-like characteristics. These detergent characteristics of NSAIDs are vital in their communication with the surface membrane phospholipids and mediate their degree of internalization into the cells and the level of intracellular ion entrapping. From the isolated mitochondria and a variety of cells, it has been demonstrated that NSAIDs amass in gastric epithelial tissues by means of ‘ion trapping’, followed by the uncoupling of mitochondrial oxidative phosphorylation and suppression of the electron transport chain. Therefore, the development of antioxidant–NSAID chimeras at a free carboxylic group of NSAIDs offers an opportunity to tackle the problems associated with a free acidic group and ROS production.

COX2 is an important membrane glycoprotein that was found to be over-expressed in inflammatory cells such as macrophages and is involved in the biocatalysis of arachidonic acid to prostaglandins required to initiate the inflammatory response [38,39]. The LOXs catalyze the synthesis of matching hydroperoxides from PUFAs such as linoleic acid and arachidonic acid. LOX enzymes are found in immunological, epithelial, and tumor cells and they perform a range of roles in the body, including inflammation, skin disorders, and carcinogenesis. 15-LOX2 was generally observed in both normal mammary epithelial cells and vascular endothelium cells. 15-LOX2 is found in various tissues, including the prostate, lung, cornea, liver, colon, kidney, spleen, ovary, and brain, but not in leukocytes. The metabolic product of 15-LOX2 is 15-hydroxyeicosatetraenoic acid (15-HETE), which is responsible for the promotion of inflammation via activation of the NF-κB pathway. 5-LOX are actively involved in the biosynthesis of lipid-based inflammatory mediators such as leukotrienes from arachidonic acid [40]. In the present work, we have developed a novel *S*-naproxen–4-allyl guaiacol chimera (MAS-1696) and examined its anti-inflammatory, analgesic, and gastro-sparing properties along with its stability.

The MAS-1696 displayed anti-inflammatory potency comparable or even superior to *S*-naproxen in carrageenan-induced edema. In the current study, the in vitro stability study of MAS-1696 was performed in simulated gastric fluid to evaluate the stability of the designed compound prior to its systemic absorption, and the observed results suggest that the compound is stable enough for the gastric absorption. The pepsin is an important component of gastric juice having a proteolytic effect for better absorption and digestion of proteins. The designed compound MAS-1696 is a nonprotein in nature and is, thus, not affected by the pepsin enzyme; thus, the pepsin enzyme was not included in the in vitro simulation studies. In comparison to *S*-naproxen, MAS-1696 caused significantly less stomach damage in healthy rats. Concurrent administration of *S*-naproxen and 4-allyl guaiacol, on the other hand, resulted in stomach injury comparable to *S*-naproxen alone. This could be because 4-allyl guaiacol is a polar antioxidant with low bioavailability, whereas the chimera MAS-1696 showed a better profile than a physical mixture (Nap + 4-AG) and it may be because of the improved physicochemical properties and better binding of the pro-moiety of chimera with the COX2 enzyme. This hypothesis was supported by the molecular docking analysis of the chimera with COX1, COX2, and 5-LOX, and MAS-1696 showed a higher affinity toward COX2 than COX1 and 5-LOX enzymes. Furthermore, enzyme assays demonstrated that MAS-1696 exhibited a greater selectivity index for COX2 over COX1 than naproxen. Molecular docking studies followed by dynamic simulation of the designed ligand complex with the COX2 enzyme have revealed that the designed ligand MAS-1696 was found to be interacting with the macromolecular residues Val116, Leu352, Tyr355, Leu359, Phe518, Met522, Val523, and Trp387 via pi interactions, while residues Ala527, Tyr348, Tyr385, Ser530, Val349, and Gly526 were found to be interacting via formation of hydrogen bonds. Thus, it can be hypothesized that these interacting residues play an important role in the selective inhibition of the COX2 enzyme. It was also noted that molecular docking of MAS-1696 shows weak interaction, although the prepared compound has good inhibitory activity. This is attributed to the fact that the designed chimera has a much higher affinity for the concerned COX2 enzyme irrespective of the fewer observed interactions as compared to the S-naproxen, although it shows a much higher anti-inflammatory response in comparison to the standard drug. The reason for such an observation is supposed to be the intensity of the observed interactions for MAS-1696, which was much higher in comparison with the interactions of the standard drug S-naproxen. Thus, the intense interaction with the important residues by the designed chimera MAS-1696 results in a selective as well as a high inhibitory effect against the COX2 enzyme. This leads to an anti-inflammatory effect free from gastric toxicity. Further, the enzymatic assay performed for the designed chimera MAS-1696 clearly suggests that it has more affinity for the COX2 enzyme as compared to that of the COX1 enzyme. However, the standard drug S-naproxen has more affinity for the COX1 enzyme when compared with the COX2 enzyme. Although the difference in the observed IC_50_ value is small, it is adequate enough to avoid the chances of COX1-mediated depletion of the protective mucous layer, causing gastrotoxicity.

For the development of a chimera as a drug candidate, it is vital to have optimum solubility and bioavailability; the chimera should remain stable enough in an acidic environment and bio-labile to be hydrolyzed back into the parent drug moieties once absorbed [9]. The stomach absorption of the majority of drugs relies on passive transport and, therefore, banks on the collective attributes of their solubility in aqueous and organic phases. Based on the existing literature, drugs displaying log *p* values ≤ 5 are supposed to be well absorbed and the chimera MAS-1696 showed a log P of 4.70 ± 0.26. For superior absorption from the GIT, the drug should be chemically stable in the simulated gastric fluid at a pH of 2–3. To evaluate this parameter, a hydrolysis study was performed in the HCl buffer of pH 2.0. The hydrolysis rate was monitored using HPLC and MAS-1696 was found to be 77% unhydrolyzed at the end of 12 h. On the other hand, MAS-1696 was readily hydrolyzed in 80% human plasma to release the parent drug molecules, which makes it desirable for oral delivery. The MAS-1696 showed highly crystalline morphology, which is an important parameter for its further utilization and development as an oral formulation.

## 4. Materials and Methods

### 4.1. Computational Studies

#### 4.1.1. Ligand Preparation

The coordinates of naproxen were imported from the PubChem database. MAS-1696 was manually drawn using ChemDraw software. The complexed ligands were detached from all the macromolecular complexes in order to obtain the nascent receptor and ligand. The nascent receptor and isolated ligand molecule were saved in default Autodock (*.pdbqt) format for redocking them to validate the utilized docking protocols [41,42,43,44,45,46].

#### 4.1.2. Macromolecular Target Selection and Optimization

The current experimental work considered protein macromolecules that have been documented for their active engagement in the inflammatory response to find the anti-inflammatory potential of the proposed ligand. COX1, COX2, 15-LOX2, 5-LOX, and the retinoic-acid-related orphan receptor (RORγ) were chosen as macromolecular targets. All the target receptors considered in the present study were set for performing docking studies by the addition of polar hydrogens, the assigning of the autodock-4 (AD4) atom type, and the assigning of Gasteiger charge and its equal distribution among all the atoms. Gridbox was prepared by overlapping every extended conformation of the ligand as well as the active macromolecular residues found to be interacting with the ligand. The grid dimensions for all macromolecular targets considered in the current research are described in Table 5.

#### 4.1.3. Molecular Docking

Molecular docking of the designed ligand was performed against various drug targets actively involved in the inflammatory reactions within the human body. Three-dimensional structures of all the target receptors considered in the current study such as COX1 (PDB id: 3kk6), COX2 (PDB id: 5ikr), 5-LOX (PDB id: 6n2w), 15-LOX2 (PDB id: 7laf), and RORγ (PDB id: 6q7a) were procured from the RCSB protein databank.

Validation of the utilized docking protocols was executed by evaluating the observed binding energy, chemical resemblance, and overlay of the docked conformation of the reference ligand with respect to its crystallized conformation [43]. After successful validation of the docking protocol, similar parameters were further utilized for docking the designed ligand against each of the considered drug targets.

### 4.2. Molecular Simulation Dynamics

Molecular simulation dynamics were performed with the intent to validate docking results by observing the stability of the drug–receptor complex with time. The complex of the designed conjugate ligand against the COX2 receptor was selected for performing dynamic simulation analysis based on their docking score. The dynamic simulation was performed for the said complex for a time period of 100 ns at a constant temperature of 300 K and constant pressure conditions [44,45,46,47,48,49].

### 4.3. Pharmacokinetic and Toxicological Profiling

The designed conjugate-based ligand requires the possession of specific physicochemical parameters to execute long-lasting medicinal effects within the human body. Estimating the ADME characteristics (absorption, distribution, metabolism, and excretion) of a specific chemical is essential throughout the drug development process. Computer simulations are reliable for the prediction of the pharmacokinetic profile of a novel ligand as they can serve as an alternative to biological studies and help reduce the use of animals in drug research, but they cannot completely replace them. Based on Lipinski’s rule, the pkCSM webserver (http://biosig.unimelb.edu.au/pkcsm/prediction; Accessed on 12 June 2022) was used to calculate important physicochemical, pharmacological, and drug-like characteristics of considered chemical substances. The designed lead molecule as well as the standard drug *S*-naproxen has been evaluated for their pharmacokinetic profile by using the pkCSM web server [50].

### 4.4. Chemical Synthesis of MAS-1696

**Scheme 1:**
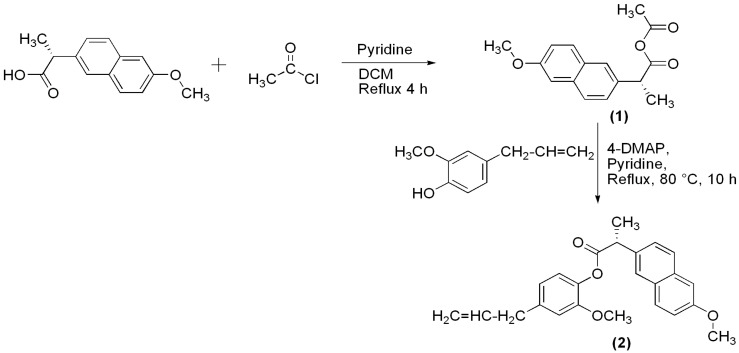
Steps involved in the synthesis of MAS-1696 (**2**).

The reactions were monitored by TLC, using silica gel 60 F_254_ as an adsorbent. The purification of MAS-1696 was carried out in silica gel (100–200 mesh) using adsorption chromatography as an adsorbent phase. A digital melting point apparatus was used to access the melting point of MAS-1696 and was uncorrected. The PerkinElmer FR-IR spectrometer was used to record the spectrum by using potassium bromide pellets. A Bruker AVANCE II 400 NMR was used to carry out ^1^H NMR and ^13^C NMR spectra at 400 and 100 MHz. The Q-Tof micro Mass Spectrometer (Waters Corporation, Milford, MA, USA) was used to obtain the ESI-MS spectrum of the MAS-1696. This Q-Tof micro Mass Spectrometer uses electrospray ionization at 70 eV. The MAS-1696 was further subjected to elemental analysis on a 2400 CHN analyzer (PerkinElmer, Waltham, MA, USA) and values were within ±0.04% of the theoretical values. Powder XRD of MAS-1696 was performed on a PANalytical X’Pert PRO diffractometer. The HPLC analysis was carried out on a Waters HPLC system with a PDA detector, and empower software system 2.1 (Figure S6). The steps involved in the synthesis of *S*-naproxen–4-allyl guaiacol chimera (MAS-1696) are described in Scheme 1.

#### 4.4.1. Synthesis of Acetic (S)-2-(6-Methoxynaphthalen-2-yl)propanoic Anhydride

The equimolar amounts of *S*-naproxen, acetyl chloride, and pyridine were allowed to reflux (for 4.5 h) in chloroform. This reaction was monitored by TLC using pet ether (60–80 °C): ethyl acetate—4:1 as a solvent system. Chloroform (50 mL × 4) was used to wash and concentrate the reactant under reduced pressure and a temperature of 60–65 °C. This treated crude anhydride product was used in the subsequent steps without any purification.

#### 4.4.2. Synthesis of (S)-4-Allyl-2-methoxyphenyl 2-(2-methoxynaphthalen-6-yl)propanoate

The anhydride derivative of *S*-naproxen and 4-allylguaiacolin equimolar amounts were refluxed at 80 °C for 10.5 h with pyridine and 4-DMAP. This reaction was monitored by TLC using the solvent system, pet ether (60–80 °C): ethyl acetate– 4:1 as a mobile phase. The reactants were treated with 10% HCl followed by chloroform extraction of the precipitated product. This product was washed four times using 10% HCl (50 mL × 4). The solvent layer was distilled off and the residue was subjected to column chromatography and again recrystallized in absolute ethanol to give the purified product **2** with a yield of 41%, mp 99–100 °C. Anal. calced. for C_24_H_24_O_4_ (376.17): % C, 76.57; H, 6.43; Found C, 76.60; H, 6.42. IR (KBr, ν cm^–1^): 3059.67 (C=CH-Ar stretch), 3008.91 (CH=CH_2_ stretch), 2974.99, 2936.95, 2841.81(Ar C–H stretch), 1750.22 (ester, C=O), 1604.83, 1508.33 (C=C stretch), 1163.20, 1147.37, 1031.40, 1000.40 (C–O–C stretch).^1^H NMR (CDCl_3_,δ ppm): 7.7910–7.7935 (1H, d, *J* = 1.00 Hz, C-7-Ar-H of nap), 7.7165–7.7491 (2H, m, C-12-H and C-5-Ar-H of nap), 7.5080–7.5338 (1H, dd, *J* = 8.56 and 1.8 Hz, C-13-Ar-H of nap), 7.1560–7.1623 (1H, d, *J* = 2.52 Hz, C-8-Ar-H of nap), 7.1347 (1H, s, C-10-Ar-H of nap), 6.8225–6.8423 (1H, broad singlet, *J* = 7.92 Hz, C-6′-Ar-H of 4-AG), 6.6863–6.7236 (2H, m, C-3′-H and C-5′-Ar-H of 4-AG), 5.8742–5.9753 (1H, m, C-8′-H of 4-AG (CH_2_–CH=CH_2_)), 5.0410–5.0929 (1H, m, 2H, C-9′-H of 4-AG (CH_2_–CH=CH_2_)), 4.1020–4.1554 (1H, q, C-2-H of nap (-CH-CH_3_)), 3.9197 (3H, s, C-14-H of nap (O–CH_3_)), 3.6580 (3H, s, C-10′-H of 4-AG (O–CH_3_)), 3.3292–3.3458 (2H, d, *J* = 6.64 Hz, C-7′-H of 4-AG (-CH_2_–CH=CH_2_)), 1.6782–1.6959 (3H, d, *J* = 7.08 Hz, C-3-H of nap (CH-CH_3_)). ^13^ C NMR (CDCl_3_,δ ppm): 172.90 (C-1), 157.64 (C-9), 150.95 (C-2′), 138.87 (C-8′), 138.20 (C-1′), 137.11 (C-4′), 135.47 (C-4), 133.75 (C-11), 129.34 (C-7), 128.98 (C-6), 127.06 (C-12), 126.51 (C-5), 126.25 (C-13), 122.33 (C-6′), 120.62 (C-5′), 118.94 (C-8), 116.08 (C-9′), 112.79 (C-3′), 105.61 (C-10), 55.73 (C-10′), 55.33 (C-14), 45.35 (C-8′), 40.06 (C-2), 18.84 (C-3). ESI-MS (*m/z*): 394.0 (M+H+NH_3_)^+^, 185.1 (100%) (C_13_H_13_O^•^).

### 4.5. Enzyme Assays

#### 4.5.1. Cyclooxygenase (COX1 and COX2) Inhibitory Assay

To evaluate the potency of MAS 1696 to inhibit iso-enzymes, COX1/2 was performed by using an ovine/human COX inhibitor assay kit. Either COX1 or COX2 (10 µL) was added to 0.1 M Tris–HCl buffer and MAS-1696 was incubated at 37 °C for 15 min at different known concentrations. After that, 10 µL of arachidonic acid (100 µM), after 2 min of 1 M HCl of 50 µL and Ellman’s reagent, was added. The absorbance at 410 nm was measured spectrophotometrically against the blank [51]. The selectivity index was calculated as follows:

SI = IC_50_ COX1/IC_50_ COX2

#### 4.5.2. 5-Lipoxygenase Inhibitory Assay

The MAS 1696 was tested against human recombinant 5-LOX using a 5-LOX assay kit. Briefly, 5-LOX solution (90 µL) was added with various MAS-1696 concentrations. The arachidonic acid (10 µL) was further added and the absorbance was measured at 490 nm against the blank [52].

### 4.6. Rat Paw Edema Test Induced by Carrageenan

In vivo studies were performed as per the guidelines issued by the WHO, Geneva. The protocol was approved by the IAEC for animal handling. The Wistar rats (male; 225–250 g) and ICR mice (20–25 g) were procured from the Animal House facility of Panjab University Chandigarh. The anti-inflammatory activity of MAS-1696 was determined by injecting carrageenan in rat paws as published by us previously [9]. The different groups of rats with six animals in each group were administered the drug. The fresh solution of carrageenan (Type IV, 0.1 mL, 1%) was used to induce acute edema by injecting it under the plantar aspect of the left hind paw. One milliliter (0.9%) of saline was administered to the right paw (control group). A plethysmometer was used to measure the change in the paw volume between two and four hours after the carrageenan challenge [9]. The rate of change in paw volume (%) was determined and demonstrated as the amount of inflammation.

### 4.7. Analgesic Activity

The ICR mice were used to carry out the analgesic activity by using the abdominal writhing assay method as described by us previously [9]. The mice were divided into six various groups (n = 6) and the writhing response was initiated by intraperitoneal (i.p.) administration of a recently formulated acetic acid solution (1%, 10 mL/kg, i.p.). The number of writhes developed after the administration of acetic acid was considered an antinociceptive response and the count of total writhes per animal was also noted in a 20 min period [9]. After 3 min of administration of the acetic acid solution, writhes were counted.

% Inhibition = [1 − Nt/Nc] × 100

where Nc—number of writhes in the control group; Nt—number of writhes in the drug-treated group.

### 4.8. Determination of Ulcer Index

The fasting rats were separated into four groups (n = 6). Animals were treated with 0.5% CMC (control), naproxen (120 mg/kg, p.o.), and equimolar doses of MAS-1696 or *S*-naproxen + 4-allyl guaiacol physical mixture. After 4 h of treatment, animals were sacrificed and the stomach was separated. This removed stomach was opened along the greater curvature and cleaned using saline to check for ulcers [9]. The ulcers were graded as:

0—Normal texture stomach

0.5—Red coloration

1.0—Spot of ulcers

1.5—Hemorrhagic streaks

2.0—Ulcers up to 3

3.0—Ulcers above 3

### 4.9. HPLC Studies

The chromatographic purity of the compound MAS-1696, partition coefficient, chemical stability, and metabolic stability were assessed using RP-HPLC. An isocratic elution system of acetonitrile-methanol-water-acetic acid (70:18:12:0.01) was used. An injection volume of 10 µL and flow rate of 1 mL/min with a relative standard deviation of 0.5% reproducibility was maintained.

### 4.10. Determination of Partition/Distribution Coefficient (Log P) of MAS-1696

The partition or distribution coefficient of MAS-1696 was observed in 1-octanol–0.05 M phosphate buffer of pH 7.4 at 25 °C. The aqueous phase and 1-octanol were stirred overnight. The MAS-1696 in 1-octanol (2 mL) and phosphate buffer (5 mL) was stirred at 25 °C in a water bath shaker for 8 h. Both layers were separated and filtered through Millipore’s 0.22 µm nylon membrane filter. The 1-octanol layer was diluted appropriately before being injected into the HPLC. The log P was determined by using the following equation:

log *P*_oct_/_wat_ = log ([solute]_octanol_)/([solute]_unionized water_)

### 4.11. In Vitro Stability of MAS-1696 in Simulated Gastric Fluid

The HCl buffer (pH 2) at 37 °C was used to evaluate the chemical stability of MAS-1696. To a 400 µL solution of MAS-1696 (10 mg/mL in chloroform), 3.6 mL of HCl buffer (pH 2) was added and kept on a shaker plate at 37 °C. Aliquots (400 µL) collected at different time points were mixed with 1600 µL of acetonitrile. After centrifugation, the supernatant was subjected to HPLC analysis. The remaining percentage of MAS-1696 was determined using the formula:

% remaining = (peak area at the respective time (min)/peak area at 0 min) × 100

### 4.12. In Vitro Human Plasma Stability of MAS-1696

To a 50 µL solution of MAS-1696 (10 mg/mL in chloroform), 900 µL of human plasma was added and kept on a shaker plate at 37 °C. Aliquots (100 µL) were collected at different intervals and mixed with 900 µL of acetonitrile for precipitation of proteins. After centrifugation, the supernatant was subjected to HPLC analysis. The remaining percentage of the conjugate was calculated using the formula:

% remaining = (peak area at the respective time (min)/peak area at 0 min) × 100.

### 4.13. Statistical Analysis

The data are expressed as the mean ± SD. One-way ANOVA and Tukey’s GraphPad Prism 6.0. multiple comparisons were used, and *p* < 0.05 was considered as statistical significance.

## 5. Conclusions

In conclusion, the research provides evidence for the promising anti-inflammatory and gastro-sparing properties of the *S*-naproxen–4-allyl guaiacol chimera (MAS-196). Computational observations are perfectly streamlined over the experimental in vitro as well as in vivo observations. In terms of stomach damage, MAS-1696 was significantly safer than S-naproxen in healthy animals; yet, with in vivo assessments of inflammation and analgesic effectiveness, it performed equally or better than S-naproxen. Notably, MAS-1696 was orally active, bio-labile, and crystalline, which further adds to it being a suitable candidate as an oral formulation. Taken together, the MAS-1696 appears a promising substitute to the existing NSAIDs for the treatment of inflammation without causing gastric toxicity.

## Figures and Tables

**Table 1:** Docking score of designed conjugate ligand and standard drug naproxen against various inflammatory drug targets.

S. No	Name	Structure	15-LOX2 (7laf)	5-LOX (6n2w)	RORγ (6q7a)	COX2 (5ikr)	COX1 (3kk6)
1	MAS-1696	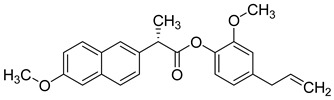	−6.17	−5.23	−8.86	−9.20	−5.2
2	Naproxen (Standard)	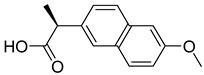	−5.67	−5.93	−6.88	−7.73	−6.21
3	Reference Ligand	Co-crystalized ligands	−7.48	−4.08	−11.30	−7.74	−10.7

**Table 2:** Inhibition percentage and IC_50_ values of MAS-1696 against cyclooxygenase and 5-lipoxygenase enzymes.

Sample	IC_50_ COX1 (µg/mL)	IC_50_ COX2 (µg/mL)	IC_50_ 5-LOX (µg/mL)	Selectivity Index
MAS-1696	3.20 ± 0.10	2.92 ± 0.18	10.72 ± 0.16	1.1
Naproxen	3.92 ± 0.02	4.62 ± 0.12	8.40 ± 0.12	0.85

(*n* = 3) and the values were presented as mean ± SD.

**Table 3:** Aqueous solubility, partition coefficient (log P), and chemical stability of MAS-1696.

Compd.	Solubility (µg/mL) (pH 7.4)	Partition Coefficient (log *P*, pH 7.4) ^a^	% MAS-1696 Left in the Simulated Gastric Fluid of pH 2				
			0 h	2 h	5 h	8 h	12 h
MAS-1696	BDL	4.70 ± 0.26	100	96.16	90.72	83.30	77.03

BDL; below the detection limit in HPLC, determined in the aqueous buffer of pH 7.4. ^a^ Results are reported as the average of two experiments (*n* = 2).

**Table 4:** Metabolic stability of MAS-1696 in human plasma.

Compd.	% MAS-1696 Left in Human Plasma		
	0 min	30 min	60 min
MAS-1696	100	0	0

**Table 5:** Coordinates used to prepare grid-box for various macromolecular targets involved in the current computational paradigm.

PDB Id	x-Axis	y-Axis	z-Axis	Spacing (Ả)	x Center	y Center	z Center
7laf	40	40	44	0.564	41.06	3.702	517.661
6n2w	40	40	40	0.597	35.944	65.322	38.344
6q7a	40	40	40	0.597	5.25	−34.743	−9.899
5ikr	46	44	46	0.592	38.042	2.131	61.28
3kk6	40	40	44	0.564	-32.531	43.584	−6.345

## Data Availability

Data are contained within the article.

## Supplementary Materials

The following supporting information can be downloaded at: https://www.mdpi.com/article/10.3390/molecules27206905/s1, Figure S1: FT-IR spectrum of MAS-1696 (**2**); Figure S2: ^1^H NMR spectrum of MAS-1696 (**2**) in CDCl_3_; Figure S3: ^13^C NMR spectrum of MAS-1696 (**2**) in CDCl_3_; Figure S4: ESI-MS spectrum of MAS-1696 (**2**); Figure S5: Powder XRD pattern of MAS-1696 (**2**); Figure S6: HPLC chromatogram of MAS-1696 (**2**); Table S1: ^1^H and ^13^C NMR interpretation of synthesized *S*-naproxen–4-allylguaiacol chimera MASS-1696 (**2**); Table S2: Powder XRD results of MAS-1696 (**2**); Table S3: 15-LOX2; Table S4: Physicochemical and toxicological profiling of designed ligand and naproxen.
