# Supplementary material for: Computational Design, Synthesis, and Pharmacological Evaluation of Naproxen-Guaiacol Chimera for Gastro-Sparing Anti-Inflammatory Response by Selective COX2 Inhibition
Source: Molecules. 2022 Oct 14;27(20):6905. doi: 10.3390/molecules27206905 (PMC9609004; doi:10.3390/molecules27206905)
Supplement: Supplementary file 1 [file molecules-27-06905-s001.zip › molecules-1953609-supplementary.pdf]

molecules

MDPI

Article

Anti-Inflammatory Activity of Naproxen-Guaiacol Chimera: Pharmacological Evaluation, Molecular Dynamics, and Predicted Toxicity

Pottathil Shinu [1,*], Manu Sharma [2,*], Giridhari Lal Gupta [3], Somdutt Mujwar [4], Mahmoud Kandeel [5], Manish Kumar[6], Anroop B. Nair [7], Manoj Goyal [8], Purna Singh [9], Mahesh Attimarad [7], Katharigatta N. Venugopala [7,10], Sreeharsha Nagaraja [7,11], Mallikarjun Telsang [12], Bandar E Aldhubiab [7] and Mohamed A. Morsy [7,13]

1 Department of Biomedical Sciences, College of Clinical Pharmacy, King Faisal University, Al-Ahsa 31982, Saudi Arabia
2 Department of Chemistry, National Forensic Sciences University Delhi Campus, New Delhi 110085, India
3 Department of Pharmaceutics, School of Pharmacy and Technology Management, SVKM's NMIMS University, Shirpur 425405, India
4 Department of Pharmaceutics, Chitkara College of Pharmacy, Chitkara University, Rajpura 140401, India
5 Department of Biomedical Sciences, College of Veterinary Medicine, King Faisal University, Al-Ahsa 31982, Saudi Arabia
6 Department of Pharmaceutics, M.M College of Pharmacy, Maharishi Markandeshwar (Deemed to be University) Ambala 133201, India
7 Department of Pharmaceutical Sciences, College of Clinical Pharmacy, King Faisal University, Al-Ahsa 31982, Saudi Arabia
8 Department of Anesthesia Technology, College of Applied Medical Sciences in Jubail, Imam Abdul Rahman Bin Faisal University, Jubail 35816, Saudi Arabia
9 Department of Physiology, College of Medicine, Saint James School of medicine, Anguilla 3872
10 Department of Biotechnology and Food Science, Faculty of Applied Sciences, Durban University of Technology, Durban 4000, South Africa
11 Department of Pharmaceutical Chemistry, Vidya Siri College of Pharmacy, Off Sarjapura Road, Bangalore 560035, India
12 Department of Surgery, College of Medicine, King Faisal University, Al-Ahsa 31982, Saudi Arabia
13 Department of Pharmacology, Faculty of Medicine, Minia University, El-Minia 61511, Egypt
* Correspondence: spottathail@kfu.edu.sa (P.S.); Tel.: +966-551732794; lantadene@hotmail.com (M.S.)

Table S1. ^{1}H and ^{13}C NMR interpretation of synthesized *S*-naproxen–4-allylguaiacol chimera MASS-1696 (**2**).

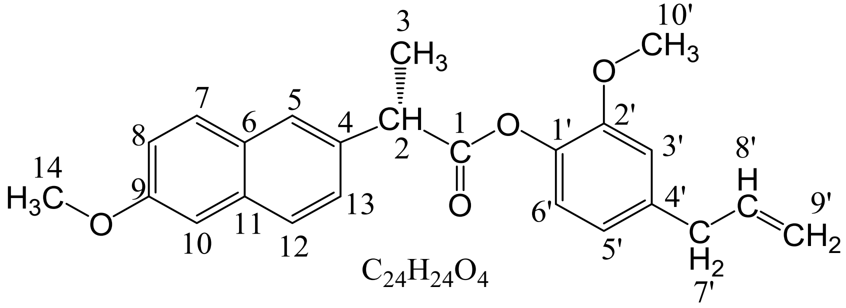

$C_{24}H_{24}O_4$

Hydrogen (s)	δ, ppm at 400 MHz	Carbon (s)	δ, ppm at 100 MHz	Carbon (s)	δ, ppm at 100 MHz
H-7	7.7910–7.7935 (1H, d, *J* = 1.00 Hz)	C-1	172.90	C-5′	120.62
H-12 & H-5	7.7165–7.7491 (2H, m)	C-9	157.64	C-8	118.94
H-13	7.5080–7.5338 (1H, dd, *J* = 8.56 & 1.8 Hz)	C-2′	150.95	C-9′	116.08
H-8	7.1560–7.1623 (1H, d, *J* = 2.52 Hz)	C-8′	138.87	C-3′	112.79
H-10	7.1347 (1H, s)	C-1′	138.20	C-10	105.61
H-6′	6.8225–6.8423 (1H, d, *J* = 7.92 Hz)	C-4′	137.11	C-10′	55.73
H-3′& H-5′	6.6863–6.7236 (2H, m)	C-4	135.47	C-14	55.33
H-8′	5.8742–5.9753 (1H, m)	C-11	133.75	C-8′	45.35
H-9′	5.0410–5.0929 (1H, m)	C-7	129.34	C-2	40.06
H-2	4.1020–4.1554 (1H, q)	C-6	128.98	C-3	18.84
H-14	3.9197 (3H, s)	C-12	127.06		

H-10′	3.6580 (3H, s)	C-5	126.51
H-7′	3.3292–3.3458 (2H, d, *J* = 6.64 Hz)	C-13	126.25
H-3	1.6782–1.6959 (3H, d, *J* = 7.08 Hz)	C-6′	122.33

Table S2. Powder XRD results of MAS-1696 (**2**).

Measurement Conditions: (Bookmark 1)

Dataset Name	EUG
File name	C:\X'Pert Data\JULY2012\EUG.xrdml
Comment	Configuration=Reflection Spinner Stage, Owner=jagtar, Creation date=12/6/2007 10:50:59 AM<br>Goniometer=PW3050/60 (Theta/Theta); Minimum step size 2Theta:0.001; Minimum step size Omega:0.001<br>Sample stage=Spinner PW3064<br>Diffractometer system=XPERT-PRO<br>Measurement program=Spinner, Owner=jagtar, Creation date=1/9/2008 11:57:34 AM
Measurement Date / Time	7/9/2012 1:08:43 PM
Operator	Panjab University
Raw Data Origin	XRD measurement (*.XRDML)
Scan Axis	Gonio
Start Position [°2Th.]	5.0084
End Position [°2Th.]	49.9904
Step Size [°2Th.]	0.0170
Scan Step Time [s]	25.1954
Scan Type	Continuous

PSD Mode	Scanning
PSD Length [°2Th.]	2.12
Offset [°2Th.]	0.0000
Divergence Slit Type	Fixed
Divergence Slit Size [°]	0.4785
Specimen Length [mm]	10.00
Measurement Temperature [°C]	25.00
Anode Material	Cu
K-Alpha1 [Å]	1.54060
K-Alpha2 [Å]	1.54443
K-Beta [Å]	1.39225
K-A2 / K-A1 Ratio	0.50000
Generator Settings	40 mA, 45 kV
Diffractometer Type	0000000011023505
Diffractometer Number	0
Goniometer Radius [mm]	240.00
Dist. Focus-Diverg. Slit [mm]	91.00
Incident Beam Monochromator	No
Spinning	No

Main Graphics, Analyze View: (Bookmark 2)

Peak List: (Bookmark 3)

Pos. [°2Th.]	FWHM [°2Th.]	d-spacing [Å]	Rel. Int. [%]	Area [cts*°2Th.]
5.3660	0.1004	16.46937	17.85	82.98
9.0120	0.1506	9.81286	68.44	477.21
10.1531	0.1338	8.71249	96.82	600.08
10.7588	0.1171	8.22327	19.16	103.90
11.7924	0.1338	7.50476	21.96	136.09
13.7934	0.1338	6.42023	45.22	280.28
14.8946	0.2342	5.94792	1.78	19.31
15.9789	0.1171	5.54669	7.04	38.18
16.1809	0.0669	5.47788	4.17	12.92
16.7101	0.0836	5.30558	22.05	85.40
16.9541	0.1004	5.22975	44.21	205.51
17.2490	0.1338	5.14102	95.42	591.35
18.0868	0.1004	4.90473	72.92	338.94
18.3634	0.1171	4.83145	29.24	158.57
18.6952	0.1004	4.74646	23.84	110.82
18.8301	0.1171	4.71276	27.90	151.28
19.0783	0.0836	4.65202	9.96	38.59
20.1505	0.1004	4.40682	15.88	73.81
20.5282	0.1004	4.32659	15.23	70.78
20.7993	0.1506	4.27081	31.65	220.65

21.6368	0.1338	4.10735	12.13	75.15
21.9301	0.1004	4.05308	12.07	56.10
22.6423	0.0669	3.92717	23.88	74.00
23.3245	0.1673	3.81384	100.00	774.70
23.5238	0.0669	3.78198	46.91	145.37
23.7278	0.0669	3.74991	24.12	74.73
24.0784	0.1171	3.69610	11.64	63.13
25.1489	0.1004	3.54115	11.88	55.21
25.4713	0.1338	3.49706	8.58	53.19
26.1536	0.1338	3.40735	8.85	54.86
26.5279	0.1673	3.36012	5.64	43.69
27.2418	0.1338	3.27367	6.57	40.75
27.7894	0.3346	3.21039	15.36	238.01
27.9888	0.1004	3.18797	11.82	54.95
28.4631	0.1338	3.13592	8.37	51.87
29.3875	0.1673	3.03934	9.26	71.70
29.6399	0.1004	3.01404	12.49	58.05
31.0445	0.1673	2.88080	6.94	53.74
32.1516	0.1673	2.78408	3.89	30.11
32.9380	0.1338	2.71939	6.10	37.81
33.4897	0.1338	2.67584	4.48	27.74
34.5796	0.3346	2.59396	2.89	44.79
35.6109	0.3346	2.52117	1.15	17.88
37.8832	0.2342	2.37501	3.03	32.90
38.7888	0.3346	2.32162	1.12	17.36
41.2369	0.2007	2.18927	2.19	20.38
42.1400	0.2007	2.14442	1.91	17.76

44.0912	0.4015	2.05394	2.00	37.27
44.8442	0.2676	2.02119	2.19	27.18
46.3187	0.4015	1.96023	1.73	32.14
47.3228	0.4896	1.91936	1.88	57.65

Table S3. Binding score and interacting residues of the designed chimera MAS-1696 against various anti-inflammatory drug targets.

Name	Structure	15-LOX2 (7laf)	5-LOX (6n2w)	ROR (6q7a)	COX2 (5ikr)	COX1 (3kk6)
MAS-1696	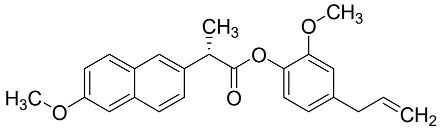	-6.17	-5.23	-8.86	-9.20	-5.2
Interacting Residues		Leu609, Phe184, Leu610, Leu420, Ile412, Asp413, His378, Ala416, Ile676, Leu415, Leu419, Ala188, Tyr185	Phe359, His600, Val604, His432, Arg596, Leu414, His372, His367, Leu368	Phe401, Ile400, Ile347, His479, Leu483, Cys393, Trp317, Cys320, Leu391, Val376, Met365, His323	Val116, Leu352, Tyr355, Leu359, Phe518, Met522, Val523, Trp387, Ala527, Tyr348, Tyr385, Ser530, Val349, Gly526	His90, Asn515, His581, Gly354, Gln350, Gln351, Gln192, Tyr94

Table S4. Physicochemical and toxicological profiling of designed ligand and naproxen.

Properties	Descriptor	MAS-1696	Naproxen
MW	(g/mol)	376.452	230.263
LogP	-	5.2946	3.0365
Rotatable bond	-	7	3
HBA	-	4	2
HBD	-	0	1
TPSA	$(\text{Å})^2$	164.902	99.642
Absorption	Water solubility (mol/L)	-6.715	-3.319
	Caco2 permeability	1.069	1.304
	Intestinal absorption (%) (human)	96.351	96.879
	Skin Permeability (Log Kp)	-2.699	-2.72
	P-glycoprotein substrate	No	No
	P-glycoprotein I inhibitor	Yes	No
	P-glycoprotein II inhibitor	Yes	No
Distribution	VDss (human)	-0.047	-0.693
	Fraction unbound (human)	0.03	0.065
	BBB permeability	0.104	0.117
	CNS permeability	-1.274	-1.4
Metabolism	CYP2D6 substrate	No	Yes
	CYP3A4 substrate	Yes	No
	CYP1A2 inhibitor	Yes	No
	CYP2C19 inhibitor	Yes	No
	CYP2C9 inhibitor	Yes	No
	CYP2D6 inhibitor	No	No
	CYP3A4 inhibitor	Yes	No
Excretion	Total Clearance (log ml/min/kg)	0.324	0.288
	Renal OCT2 substrate	No	No
Toxicity	AMES toxicity	No	No
	Max. tolerated dose (human) (log mg/kg/day)	0.485	0.71
	hERG I inhibitor	No	No

	hERG II inhibitor	Yes	No
	Oral Rat Acute Toxicity (LD50) (mol/kg)	2.57	3.055
	Oral Rat Chronic Toxicity (LOAEL) (mg/kg/day)	2.028	2.333
	Hepatotoxicity	No	No
	Skin Sensitisation	No	No
	T. Pyriformis toxicity (mg/L)	0.392	0.339
	Minnow toxicity	-1.8	-0.045

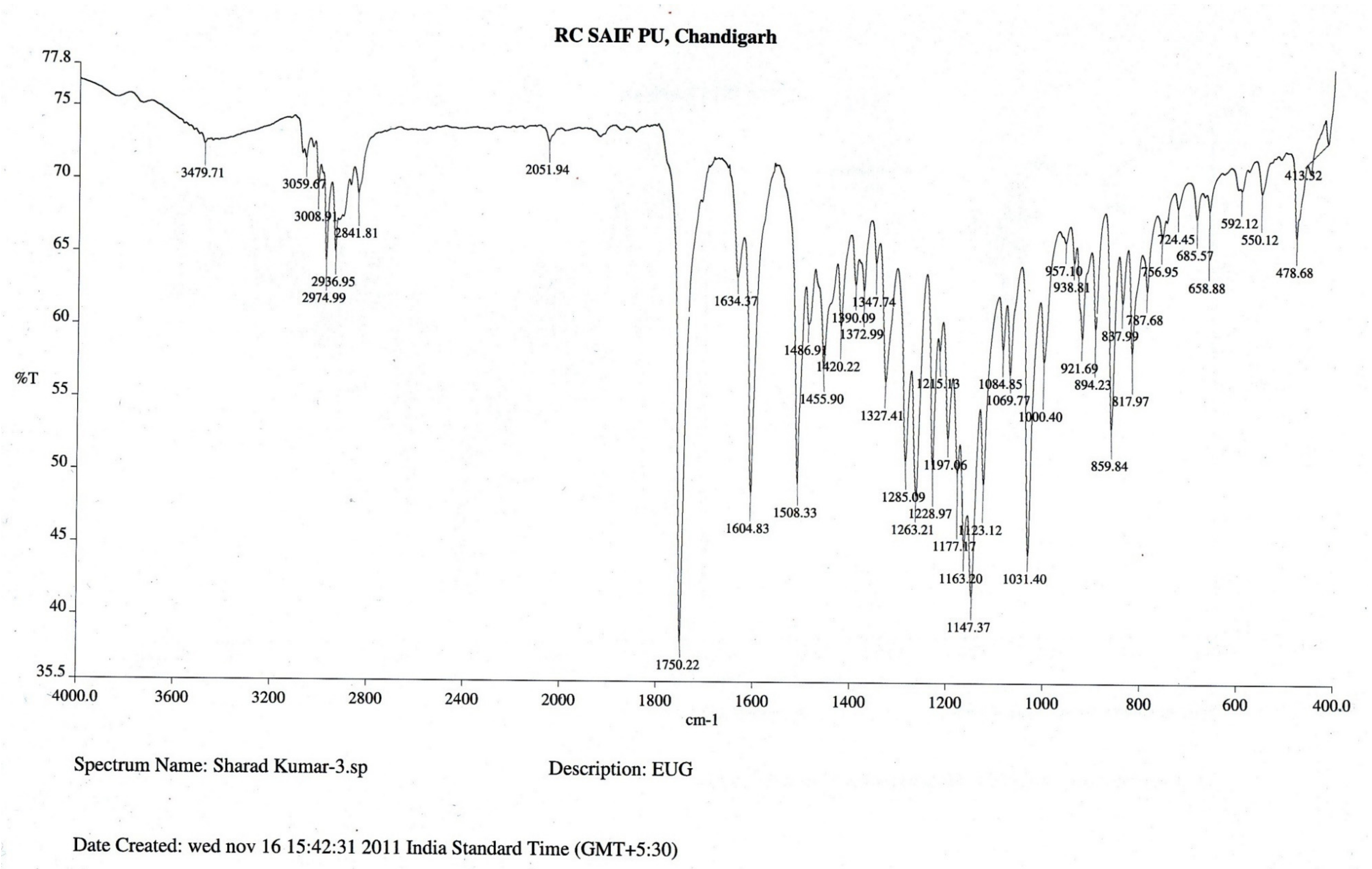

Figure S1. FT-IR spectrum of MAS-1696 (**2**).

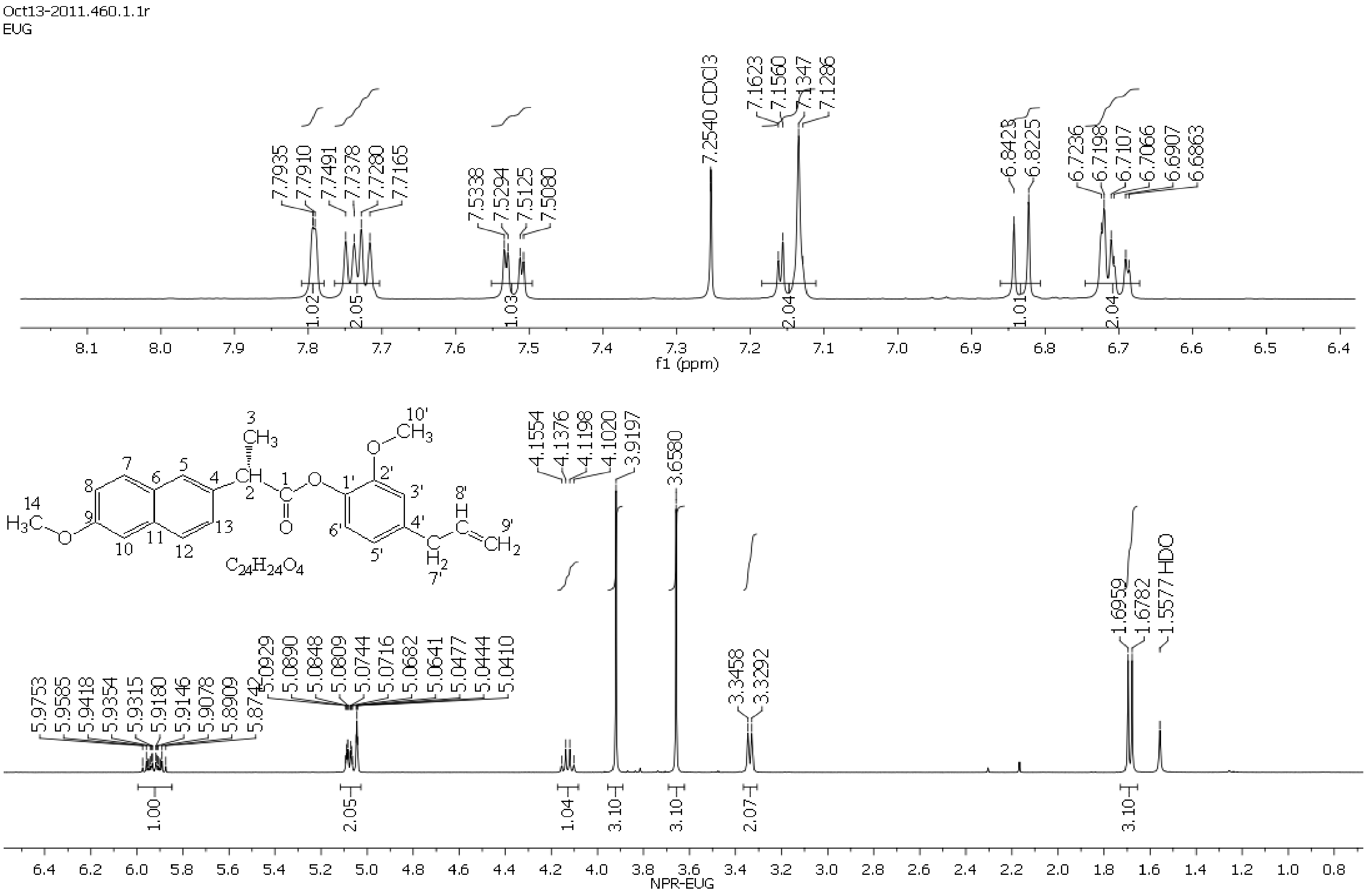

Figure S2. ^{1}H NMR spectrum of MAS-1696 (**2**) in $CDCl_3$; Molecular Formula: $C_{24}H_{24}O_4$.

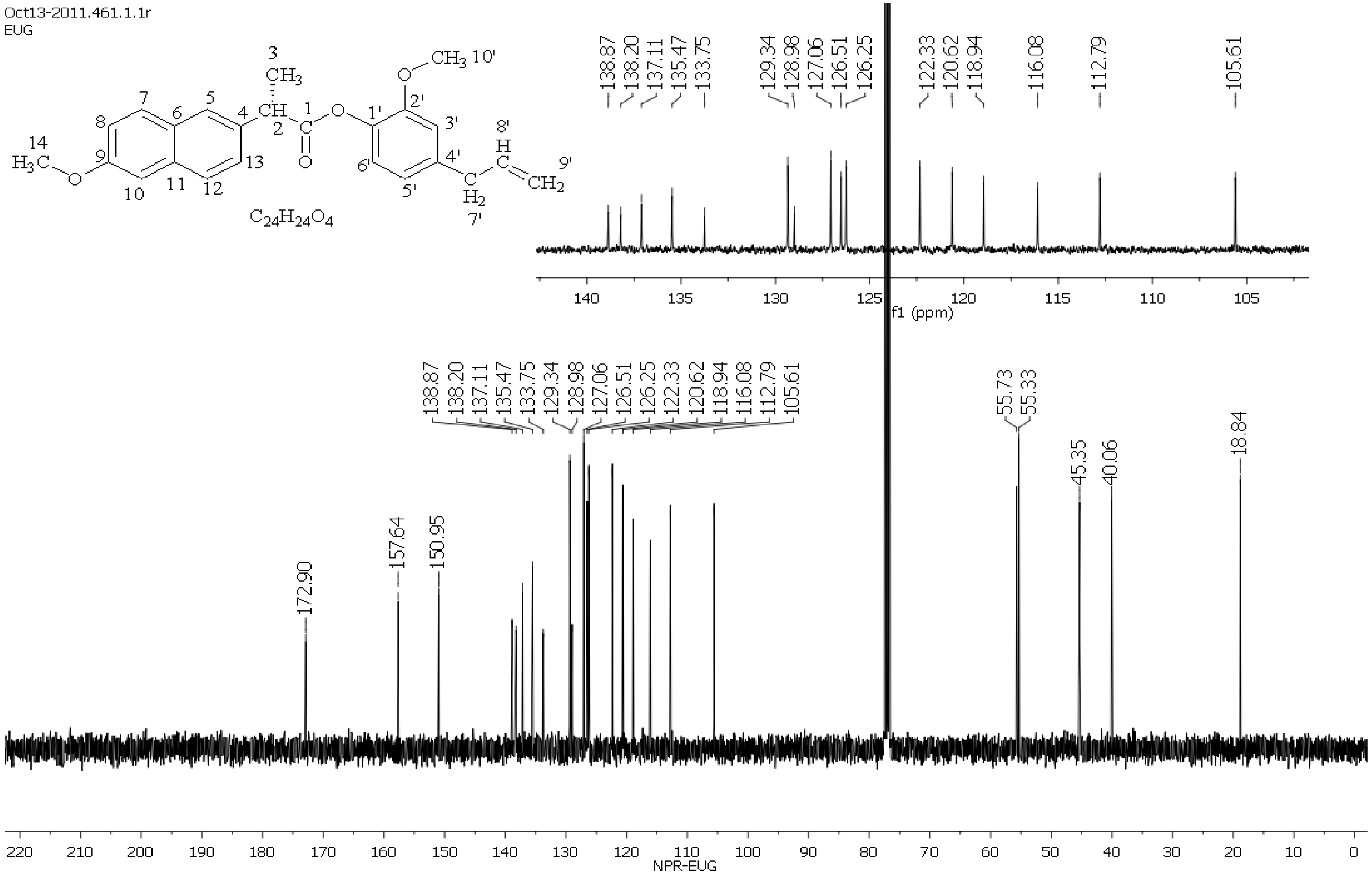

Figure S3. ^{13}C NMR spectrum of MAS-1696 (**2**) in $CDCl_3$; Molecular Formula: $C_{24}H_{24}O_4$. All the 24 carbons/peaks are visible in the spectrum.

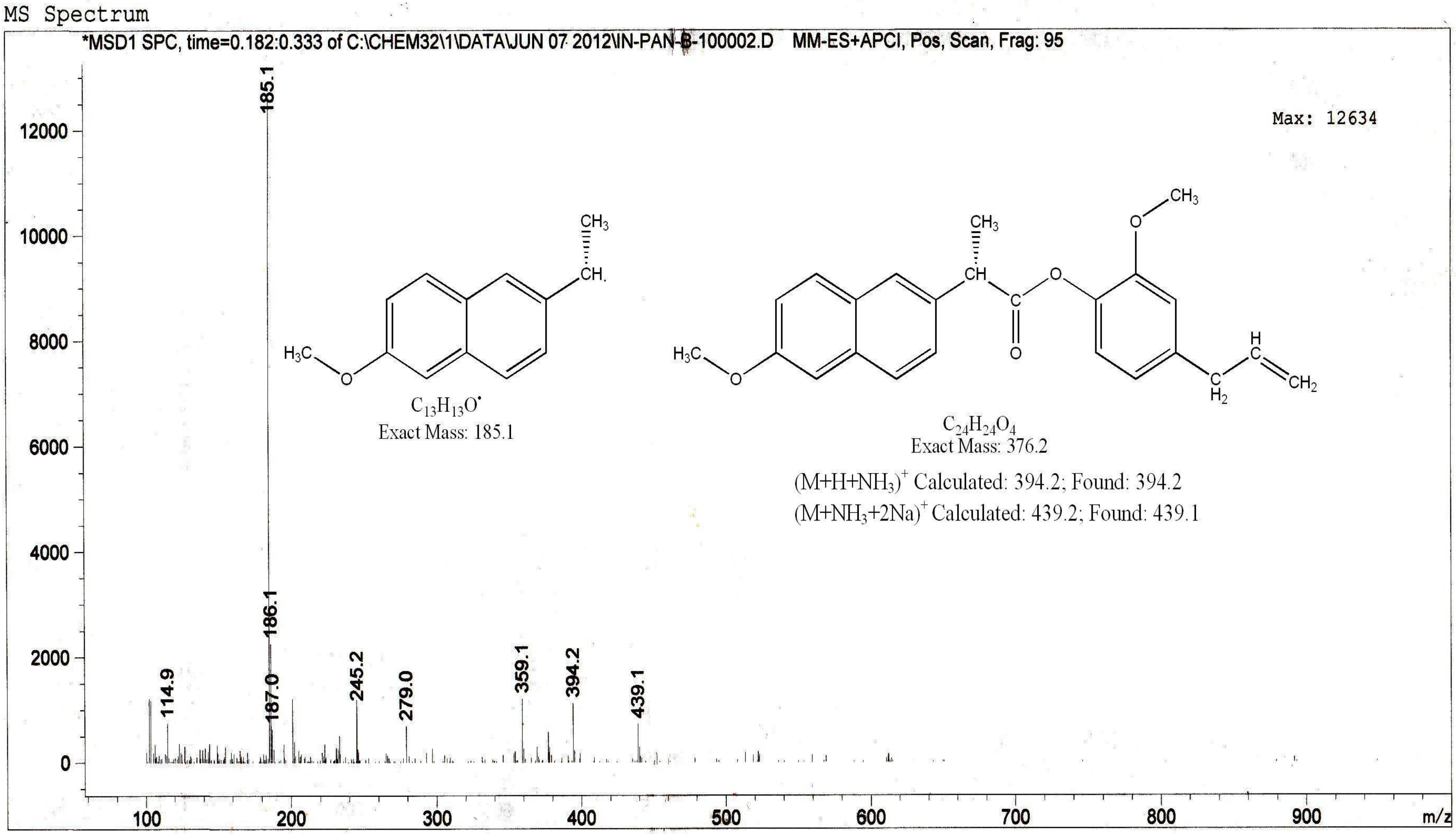

Figure S4. ESI-MS spectrum of MAS-1696 (**2**); Exact Mass $(M+H+NH_3)^+$: 394.2; Found: 394.2.

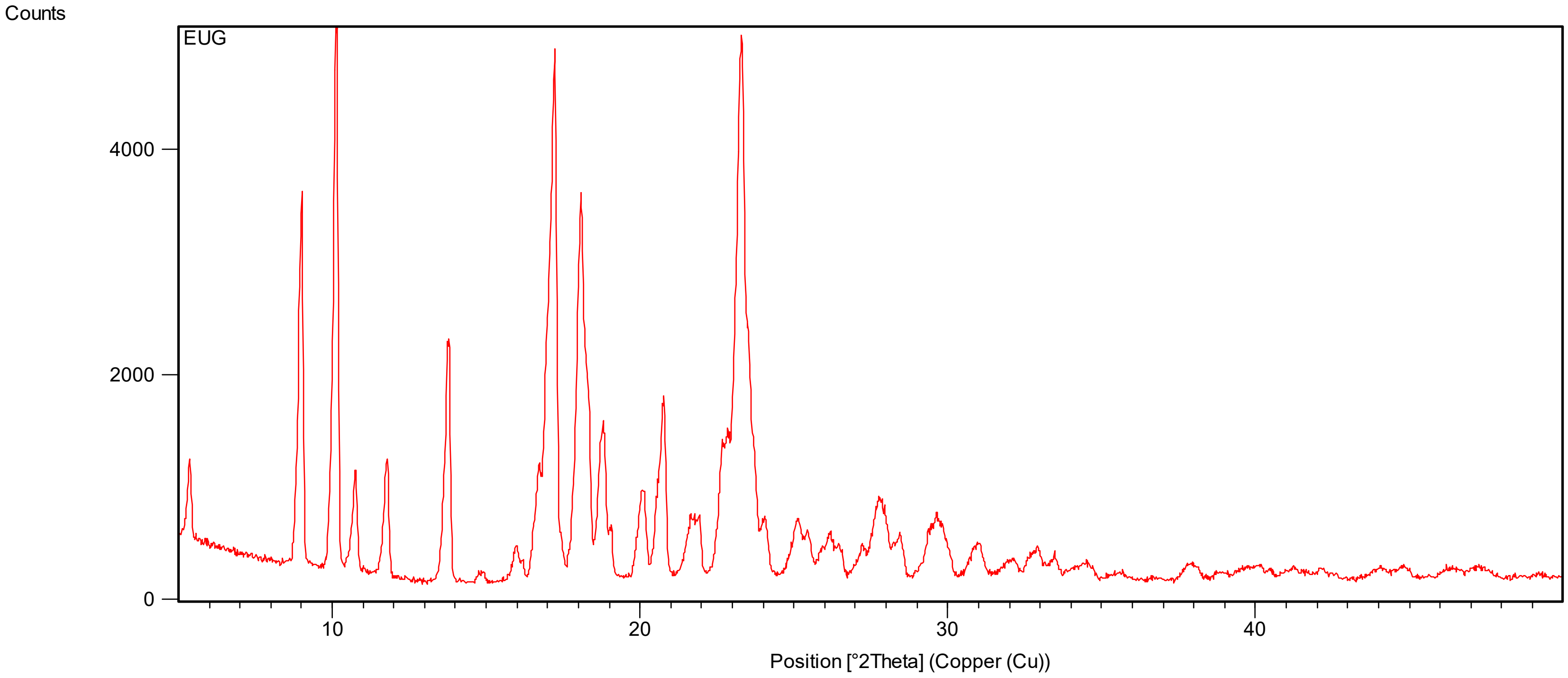

Figure S5. Powder XRD-pattern of MAS-1696 (**2**).

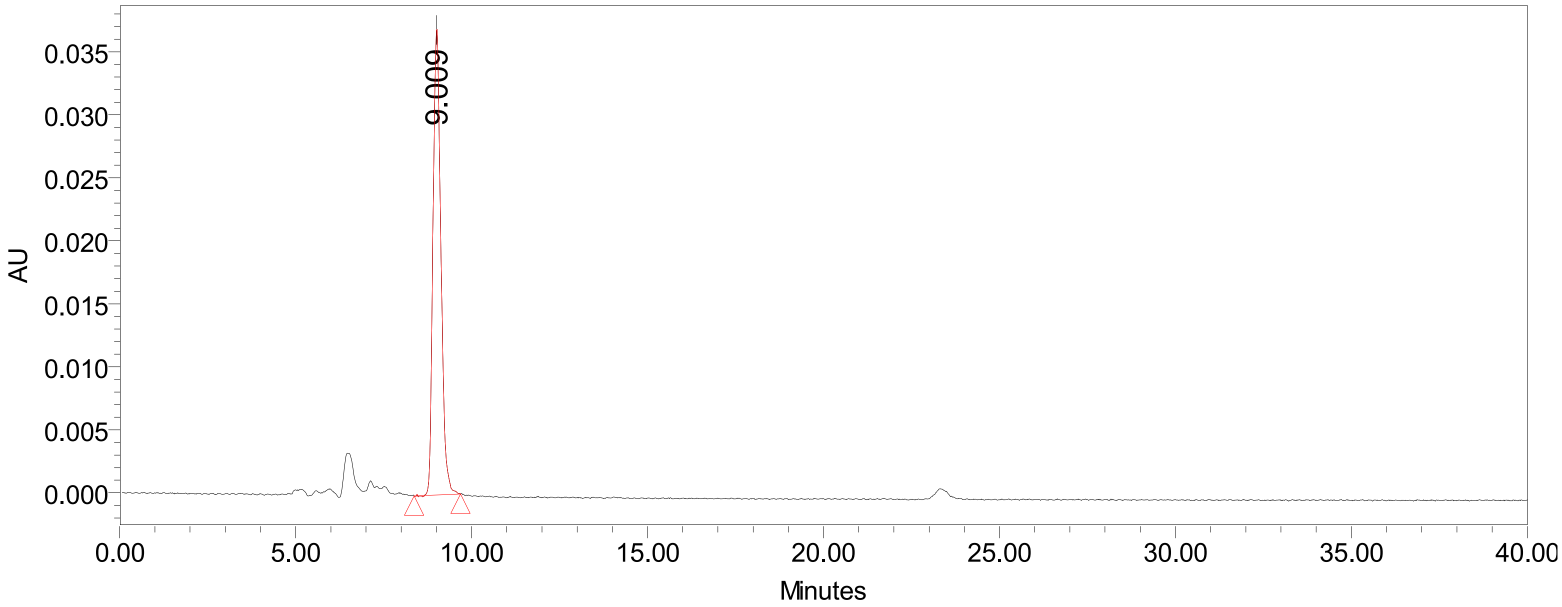

Figure S6. HPLC chromatogram of MAS-1996 (t_R 9.009 min).
